# Retraction: Percutaneous Aspiration Thrombectomy for Arterial Thromboembolism during Infrainguinal Endovascular Recanalization

**DOI:** 10.1371/journal.pone.0196768

**Published:** 2018-04-26

**Authors:** The PLOS ONE Editors

Following publication of this article [1], the authors requested its retraction due to errors in the data analyses. A member of *PLOS ONE*’s Editorial Board confirmed that the statistical analyses were not done correctly and as such the conclusions of the article have been called into question.

The specific concerns include:

1. The regression model used was not appropriate in light of the following issues:
  - The events-per-variable ratio has to be greater than 10 for the model that was applied. In this case the ratio was 4.6 (23/5).
  - The authors did not study the functional form of the continuous predictors, but rather used the linear form without explanation.
  - Discrimination and calibration were not assessed for the model.
2. The authors should have used a test for ordinal variables.
3. The authors used a t-test to analyze mTIMI flow grade and pulse volume scoring. This test was not appropriate given the nature of these variables; instead, a non-parametric test should have been applied.
4. In Table 3 the authors incorrectly interpreted the results by considering that OR>0 indicated a positive or negative predictor. Following publication, the authors consulted a statistician who advised that a cutoff of OR>1 should have been used instead. Furthermore, the high OR values and broad confidence intervals did not support conclusions including statements of statistical significance.
5. The authors grouped arterial thrombosis and distal embolism cases together instead of conducting separate analyses to identify predictors of these clinical outcomes.

In light of these issues, the *PLOS ONE* Editors retract the article, as the conclusions are not supported by the data and analyses presented.

The authors did not comment on the retraction decision.
